# Decomposing Midlife HS-GED Health Disparities: The Role of Employment Histories

**DOI:** 10.1093/geroni/igaf122.188

**Published:** 2025-12-31

**Authors:** Wenxuan Huang

**Affiliations:** Johns Hopkins University, Baltimore, Maryland, United States

## Abstract

**Background:**

The health disparities between high school (HS) diploma holders and GED recipients have been well documented. However, limited research has examined how differential employment histories may contribute the observed HS-GED health disparities.

**Data:**

This study uses data from the National Longitudinal Survey of Youth 1979 (NLSY79, n = 3,277) to construct the employment histories (age 21-49) and examine eight health outcomes (e.g., self-rated health, functional limitations and depressive symptoms, etc.) observed in the 50+ Health Module.

**Methods:**

This study employs sequence-based analysis to classify employment histories into typical life course patterns. It then uses multivariate decomposition approach to determine the extent to which the HS-GED health disparities are attributable to differential distribution or differential return of typical employment histories.

**Results:**

This study identifies six typical employment patterns: (1) gradual withdrawal, (2) incremental integration, (3) prolonged inactive, (4) continuous full-time, (5) part-time dominated, and (6) military to civil work. The decomposition analysis suggests that both differential distribution and differential return between HS and GED groups play a role in explaining the HS-GED disparities, while the relative shares differ across health outcomes. Especially, the differential distribution of “continuous full-time” and “prolonged inactive” employment histories arise to be the main drivers of the HS-GED health disparities. These findings highlight the crucial role of employment histories in shaping HS-GED health disparities and raise the question of why similar employment histories yield discriminatory health returns between these two groups.

